# MIF‐Mediated NLRP3 Inflammasome‐Dependent Pyroptosis in Spinal Neurons and Microglial Polarization Facilitate Neuropathic Pain Progression

**DOI:** 10.1155/anrp/6624776

**Published:** 2025-11-27

**Authors:** Feng Zhou, Yue Tian, Wei Liao, Qingling Ma, Han Bao, Fanqing Meng, Jingjing Jiang

**Affiliations:** ^1^ Department of Anesthesiology, Jinan Maternity and Child Care Hospital Affiliated to Shandong First Medical University, Jinan, China; ^2^ Department of Anesthesiology, Shengjing Hospital of China Medical University, Shenyang, Liaoning, China, cmu.edu.cn

**Keywords:** microglia polarization, MIF, neuropathic pain, NLRP3 inflammasome, pyroptosis

## Abstract

Despite considerable advancements in therapeutic approaches, neuropathic pain remains a globally prevalent and challenging source of chronic suffering, underscoring the urgent demand for innovative treatment strategies. Here, we investigated the role of macrophage migration inhibitory factor (MIF) in neuropathic pain using a rodent model of chronic constriction injury (CCI) to the sciatic nerve, approved by the Ethical Committee of Animal Use and Care. Mechanical thresholds (von Frey hairs) and thermal latencies (hot plate) were measured, alongside spinal MIF expression, pyroptosis markers (e.g., GSDMD‐N), and inflammatory cytokines (IL‐1β, IL‐6, and TNF‐α). Our results showed that spinal MIF levels surged post‐CCI, peaking on day 14 (*p* < 0.001), and drove microglial M1 polarization and inflammatory cytokine release (all *p* < 0.01). Notably, MIF activated the NLRP3 inflammasome, exacerbating neuronal pyroptosis (*p* < 0.01). These effects were mitigated by MIF inhibitor ISO‐1 or NF‐κB inhibitor PDTC, which reduced neuroinflammation and pain hypersensitivity. Collectively, this study reveals that MIF promotes NLRP3 inflammasome‐mediated neuronal pyroptosis and microglial polarization via the NF‐κB pathway, providing novel mechanistic insights into neuropathic pain alleviation through MIF inhibition.

## 1. Introduction

Neuropathic pain is caused by damage to or a disease of the somatosensory nervous system. Neuropathic pain arises from nervous system damage caused by tumors, diabetes, infections, autoimmune diseases, chemotherapy, or trauma [1]. Its prevalence in the general population may reach 7%–8%, accounting for 20%–25% of individuals with chronic pain [2]. Current first‐line pharmacotherapies include gabapentinoids (e.g., gabapentin, pregabalin), antidepressants (e.g., duloxetine), and topical agents (e.g., lidocaine patches), while opioids are reserved for refractory cases because of risks of dependence and hyperalgesia [3]. Other therapeutic medications include gabapentin and nonsteroidal anti‐inflammatory drugs; however, even with multimodal analgesic management, treatment effectiveness is unsatisfactory [4]. Despite significant research in areas including neuroinflammation, oxidative stress, ion channels, mitochondrial function, central sensitization, and glial cell activation, the molecular mechanisms underlying neuropathic pain remain unclear, greatly impeding the advancement of its treatment [5, 6]. Therefore, treating neuropathic pain requires improved strategies.

Cell death occurs in two main forms: necrosis and programmed cell death, in which cell death is spontaneously determined by encoded signals or genes, including those involved in apoptosis, pyroptosis, autophagy, and necroptosis [7, 8]. Pyroptosis, a form of inflammatory cell death mediated by pore‐forming proteins, is characterized by the concomitant initiation of inflammatory and immune responses [9]. Its characteristic feature is the formation of cell membrane pores by the action of pore‐forming proteins, perturbing ion concentrations on both sides of the cell membrane, resulting in cell swelling, rupture, and release of various proinflammatory factors, including IL‐1β, IL‐18, and ATP [10]. The released substances trigger inflammation outside the cell, causing a series of subsequent reactions. The inflammasome induces pyroptosis; the NLRP3 inflammasome is extensively researched [11]. Gasdermin D (GSDMD) serves as a critical pore‐forming effector that is cleaved by inflammasome‐activated caspase‐1, yielding its active N‐terminal fragment (GSDMD‐N) [12]. GSDMD‐N binds to acidic phospholipids to form pores, disrupting membrane integrity, increasing the release of mature IL‐1β and IL‐18, and leading to pyroptosis [13]. Despite many studies on the correlation between NLRP3 inflammasomes and GSDMD‐induced pyroptosis, the mechanisms underlying cellular pyroptosis in neuropathic pain remain elusive.

Macrophage migration inhibitory factor (MIF) is a proinflammatory pleiotropic cytokine with extensive immunoregulatory functions [14]. MIF is widely expressed in various neural cell types, including microglia, astrocytes, neurons, and dendritic cells, and is rapidly released in response to various stimuli [15]. MIF is involved in the pathogenesis of several inflammatory and autoimmune diseases, including sepsis, rheumatoid arthritis, and systemic lupus erythematosus [15]. MIF stimulates microglia to produce proinflammatory factors, including IL‐1β, IL‐6, and TNF‐α, through multiple signaling pathways to trigger inflammatory responses [16]. MIF mediates cell pyroptosis in sepsis‐induced acute kidney injury by regulating the NF‐κB/NLRP3/GSDMD signaling pathway [17]. These studies suggest a close association between MIF and NLRP3 inflammasome activation during pyroptosis.

However, no empirical evidence has linked macrophage MIF to the regulation of cellular pyroptosis in the context of neuropathic pain. Informed by prior research, we postulated that MIF upregulation following nerve injury exacerbates pyroptosis by promoting NLRP3 inflammasome activation via the NF‐κB signaling pathway. To test this, a neuropathic pain model was established in mice using chronic constriction injury (CCI) surgery. Our observations revealed a significant increase in MIF expression in the spinal cord post‐injury, with primary localization to spinal neurons rather than microglia or astrocytes. Functionally, MIF was found to promote microglial polarization toward an M1 phenotype, amplify the expression of inflammatory mediators, and induce the upregulation of pyroptosis‐associated proteins through NF‐κB pathway activation. This cascade ultimately enhances pyroptosis in spinal neurons, contributing to the mechanical allodynia observed in CCI mice. Conversely, administration of the MIF inhibitor ISO‐1 suppressed MIF activity, attenuating the upregulation of NLRP3 inflammasome components, M1 microglial polarization, pyroptosis‐related protein expression, and spinal levels of inflammatory factors, which collectively alleviated mechanical allodynia. Furthermore, inhibition of the NF‐κB subunit p65 with PDTC similarly reduced the expression of NLRP3‐related and pyroptosis‐related proteins, suppressed M1 microglial polarization, and decreased inflammatory factor levels in the spinal cord. In summary, this study provides compelling experimental evidence elucidating the mechanism by which MIF modulates the NF‐κB–NLRP3 axis in neuropathic pain.

## 2. Materials and Methods

### 2.1. Experimental Animals

Male C57BL/6J wild‐type mice (8 weeks old) were provided by the Animal Experimental Center of Shandong First Medical University (SDFMU) (Jinan, China). All mice were housed in standard cages under conditions of 23°C, 55% humidity, and a 12:12‐h light–dark cycle. All animal experiments were approved by the SDFMU Scientific Investigation Committee and adhered to the guidelines of the International Association for the Study of Pain.

### 2.2. Neuropathic Pain Model Induced by CCI

Anesthesia was induced in mice via intraperitoneal injection of 2% pentobarbital sodium (30 mg/kg), following which a CCI model was established according to a previously described method with minor modifications. The left sciatic nerve was carefully exposed, and four loose ligatures were tied around it. The distance between the ligatures was approximately 1 mm; equal tension was applied to all ligatures. Finally, the nerve was repositioned, and the muscle and skin layers were sutured after ligation. In the sham surgery group, the sciatic nerve was exposed without ligation.

### 2.3. Intrathecal Administration

Intrathecal catheters (PE‐10 tubing; Lake Havasu City, AZ, USA) were surgically implanted in mice for drug administration, following a previously established protocol [18]. In brief, the surgical site was shaved and disinfected, followed by a midline incision to expose the spinous processes. The paraspinal muscles were then dissected away. Under a surgical microscope, a small burr hole (approximately 1 × 1 mm) was carefully drilled to expose the dura mater, which was subsequently incised. Penetration of the dura with a 25‐gauge needle was confirmed by observable cerebrospinal fluid leakage. The implanted catheter was fixed in place with a droplet of tissue adhesive (Histoacryl, B. Braun; Tuttingen, Germany) and anchored to the paraspinal fascial layer. To prevent infection, penicillin sodium (10,000 IU, Shanghai AoBopharmtech; Shanghai, China) was administered intramuscularly postoperatively. Mice exhibiting any neurological deficits following catheterization were excluded from the study. All experimental agents were delivered intrathecally in a 5 μL volume via a microsyringe over a period of no less than 30 s, and the catheter dead space was flushed with 10 μL of saline immediately after administration.

### 2.4. Behavioral Tests

#### 2.4.1. Mechanical Hyperalgesia

Behavioral tests were conducted 60 min after intrathecal drug administration (ISO‐1/PDTC) or vehicle (DMSO). All tests were performed between 9:00 and 12:00 a.m. to minimize circadian influence. Mice were acclimated to the room environment for 30 min in a brown organic glass box on a metal wire mesh platform. Calibrated von Frey filaments were applied to the central area of the injured hind paw sole in increasing the order of bending force. Sufficient force was applied to each filament to bend for 5 s, repeated at 6‐min intervals. The cutoff value was 5.0 g to prevent injury to the animals. Withdrawal of the paw, licking, and shaking were considered nociceptive responses. The test was repeated three times with a stimulus interval of 5 min; the average of the three repeated tests was recorded as the paw withdrawal threshold (PWT). Baseline PWT was obtained 1 day before constructing the neuropathic pain model; mechanical allodynia was tested on days 7, 14, and 21 after CCI surgery.

#### 2.4.2. Thermal Hyperalgesia

The mice were acclimated for 30 min to the room environment in a transparent plastic box on a glass surface. The plantar surface of the hind paw was vertically heated using a laser with a diameter of 5 mm. When a mouse withdrew its paw, the movement was detected using an infrared heat–induced pain stimulator (RWD; China). The responses included lifting the foot and avoidance.

### 2.5. Western Blot

Lumbar spinal cord tissues from the model mice were homogenized in RIPA lysis buffer containing protease and phosphatase inhibitors (Epizyme; Shanghai, China). The homogenate was centrifuged at 12,000 × *g* for 10 min to collect the supernatant. Total protein concentration was quantified using a bicinchoninic acid (BCA) assay kit (Epizyme; Shanghai, China). Equal amounts of protein were separated by 10% sodium dodecyl sulfate–polyacrylamide gel electrophoresis (SDS‐PAGE) and subsequently transferred onto 0.2 μm polyvinylidene fluoride (PVDF) membranes (Millipore; USA) via the wet transfer method. The membranes were blocked with protein‐free rapid blocking buffer (Epizyme; Shanghai, China) for 1 h at room temperature with gentle shaking, followed by an overnight incubation with specific primary antibodies at 4°C. The primary antibodies employed in this study were as follows: anti‐GAPDH (Proteintech, 10494‐1‐AP), anti‐NLRP3 (Proteintech, 27458‐1‐AP), anti‐NF‐κB p65 (Proteintech, 66535‐1‐Ig), anti‐GSDMD (Proteintech, 20770‐1‐AP), anti‐GSDMD‐N terminal (Abcam, ab219800), anti‐IL‐1β (Proteintech, 16806‐1‐AP), anti‐TNF‐α (Proteintech, 60291‐1‐Ig), anti‐IL‐6 (Proteintech, 21865‐1‐AP), anti‐CD68 (Proteintech, 28058‐1‐AP), anti‐CD206 (Proteintech, 60143‐1‐Ig), anti‐MIF (Proteintech, 20415‐1‐AP), anti‐caspase‐1 (Proteintech, 22915‐1‐AP), and anti–Phospho‐NF‐κB p65 (Ser468) (Proteintech, 82335‐1‐RR). The following day, membranes were washed three times with Tris‐buffered saline containing 0.1% Tween‐20 (TBST, Servicebio; Wuhan, China) and then incubated with horseradish peroxidase (HRP)‐conjugated anti‐rabbit (Cell Signaling Technology [CST], 7074S) or anti‐mouse (CST, 7076S) secondary antibodies for 2 h at room temperature. Protein bands were visualized using an enhanced chemiluminescence (ECL) reagent (Simuwu; China), imaged with a Tanon gel imaging system (Tanon; China), and quantified by densitometry using ImageJ software (National Institutes of Health [NIH]; Bethesda, MD, USA).

### 2.6. Immunofluorescence

Immunofluorescence was performed on lumbar spinal cord sections (L4‐L5). At 14 days post‐surgery, CCI and sham‐operated mice under deep anesthesia induced by an overdose of pentobarbital sodium were transcardially perfused with phosphate‐buffered saline (PBS) until the effluent was clear of blood, followed by perfusion with 4% cold paraformaldehyde (PFA). The lumbar spinal cord was then dissected, post‐fixed in 4% PFA for 5 h, and cryoprotected by immersion in a 25% sucrose solution until the tissues sank. Tissues were embedded in O.C.T. compound (SAKURA; USA) and frozen at −20°C in a cryostat (Leica; Germany). Transverse sections of 20 μm thickness were prepared as previously described. For single‐ or double‐labeling immunofluorescence, sections were incubated overnight at room temperature with primary antibodies. The primary antibodies used included: anti‐MIF (Proteintech, 20415‐1‐AP), anti‐Iba1 (Servicebio, GB11105), anti‐GFAP (Servicebio, GB11096), anti‐NeuN (Servicebio, GB11138), anti‐NLRP3 (Proteintech, 27458‐1‐AP), and anti‐GSDMD (Abcam, ab219800). After incubation, sections were washed three times with TBST and incubated with appropriate fluorescent secondary antibodies, such as Cy3‐conjugated anti‐rabbit IgG (Servicebio, GB21403), for 1 h at room temperature. Following final washes, sections were coverslipped. Laminar structures (Laminae I‐IV) were identified based on established cytoarchitectonic features. All images were captured using a Nikon Eclipse E600 fluorescence microscope (Nikon; Japan).

### 2.7. Statistical Analysis

All data are presented as the mean ± standard deviation. Statistical analyses were performed using GraphPad Prism 9.0 software (GraphPad Software, Inc.; San Diego, CA, USA). Comparisons between multiple groups were conducted using one‐way analysis of variance (ANOVA) followed by Bonferroni’s post hoc test. For the analysis of dendritic arborization via Sholl analysis and time‐series data, a two‐way ANOVA with repeated measures was employed. A *p* value of less than 0.05 was considered statistically significant for all tests.

## 3. Results

### 3.1. Upregulation of MIF Expression in Neuropathic Pain

On postoperative days 7, 14, and 21, the CCI mice exhibited significant mechanical (Figure 1(a); Psham vs. CCI < 0.001; *n* = 10 in the sham group, and *n* = 10 in the CCI group) and thermal hyperalgesia (Figure 1(b); Psham vs. CCI < 0.001; *n* = 10 in the sham group, and *n* = 10 in the CCI group) compared to the Sham group mice, suggesting that the neuropathic pain model was successfully established. To analyze changes in MIF expression in neuropathic pain, we examined MIF expression in the spinal cord on postoperative days 7, 14, and 21. Compared to the Sham group, the protein expression levels of MIF in the spinal cord were significantly increased, reaching a peak on postoperative day 14 (Figures 1(c) and 1(d); Psham vs. 7 d = 0.0264; Psham vs. 14 d = 0.0005; Psham vs. 21 d = 0.0028; *n* = 3 in the sham group, and *n* = 3 in the CCI group). Consistent with the western blot results, sciatic nerve ligation significantly enhanced the mean fluorescence intensity of MIF in immunofluorescence staining (Figure 1(e)). Exploring MIF’s cellular localization in the spinal cord using immunofluorescence revealed that MIF co‐localized primarily with NeuN‐positive neurons in the spinal cord (Figure 1(f)) rather than with the microglial cell marker Iba‐1 or the astrocyte marker GFAP.

Figure 1MIF expression is increased during complete chronic constriction injury (CCI) surgery‐induced neuropathic pain. (a) MWT and (b) TWL exhibited a significant reduction following CCI surgery. (c) Representative Western blot images depicting MIF expression levels in the spinal cord dorsal horn at days 7, 14, and 21 post‐surgery. (d) Quantitative analysis of MIF protein levels normalized to a loading control, confirming its temporal upregulation. (e) Immunofluorescence analysis further corroborated the alterations in MIF expression across different experimental groups (Scale bar = 50 μm). (f) Double‐label immunofluorescence was performed to assess the cellular localization of MIF (red) with markers for microglia (Iba1), astrocytes (GFAP), and neurons (NeuN) at postoperative day 14, coinciding with the peak MIF expression. Scale bar = 50 μm. All data are expressed as the mean ± SD and are representative of three independent experiments performed with five mice per group. ^∗^ *p* < 0.05, ^∗∗^ *p* < 0.01, and ^∗∗∗^ *p* < 0.001.

**Figure:**
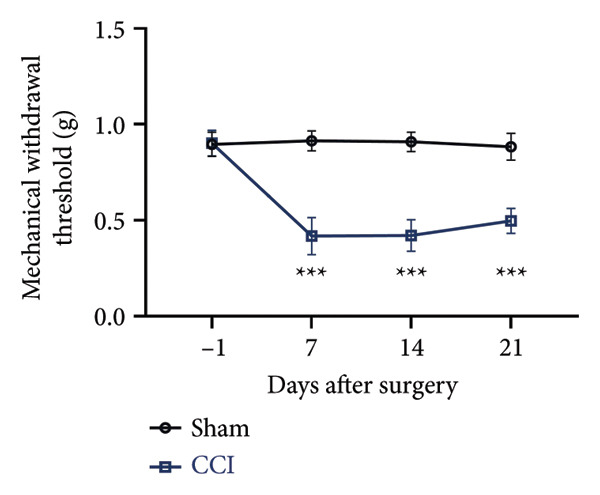
(a)

**Figure:**
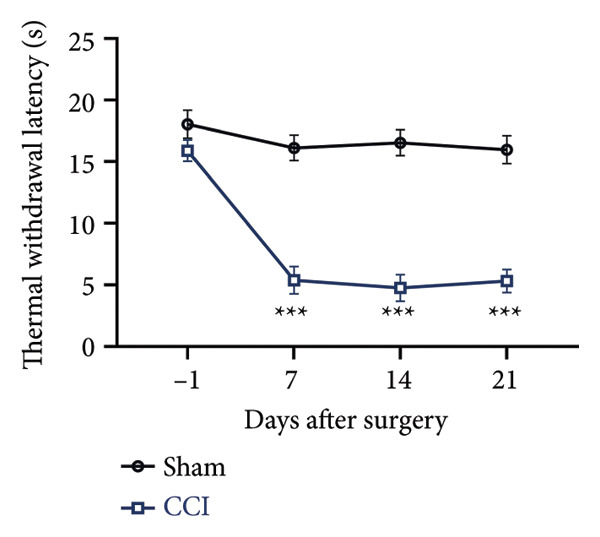
(b)

**Figure:**
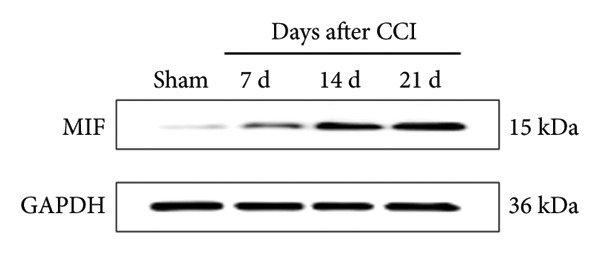
(c)

**Figure:**
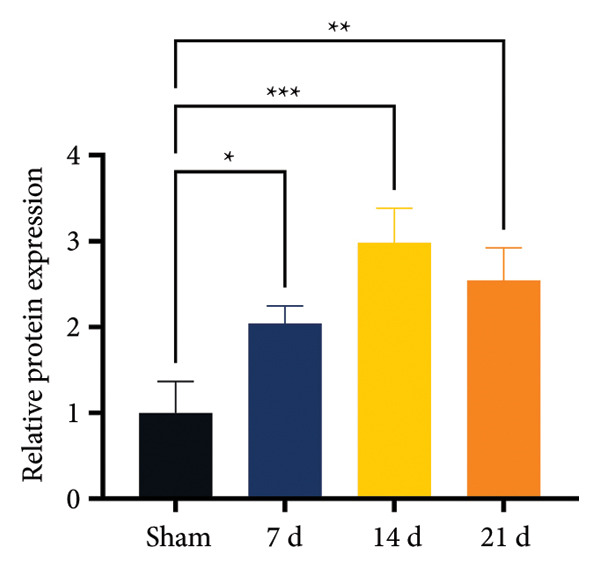
(d)

**Figure:**
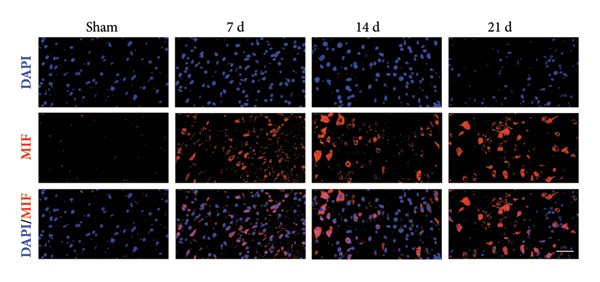
(e)

**Figure:**
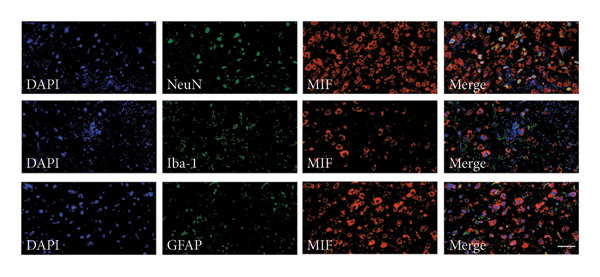
(f)

### 3.2. Neuropathic Pain Induces Microglial Polarization and Neuroinflammation Coinciding With MIF Upregulation

Because MIF promotes microglial cell activation and polarization, we measured the expression changes of Iba‐1 on postoperative day 14 using immunofluorescence. The analysis showed increased activation of microglial cells on postoperative day 14 (Figures 2(a) and 2(b); Psham vs. 14 d < 0.0001). Western blotting was used to detect changes in the expression of CD68 (an M1 phenotype marker in microglial cells) and CD206 (an M2 phenotype marker in microglial cells). Compared to the sham group mice, the expression of CD68 in the spinal cord of CCI group mice increased; the expression of CD206 showed no significant change (Figures 2(c), 2(d), and 2(e); CD68: Psham vs. 7 d = 0.0002; Psham vs. 14 d < 0.0001; Psham vs. 21 d < 0.0001; *n* = 3 in the sham group, and *n* = 3 in the CCI group). Because M1 polarization of microglial cells correlates strongly with neuroinflammation, we used western blotting to measure the protein levels of proinflammatory cytokines in the spinal cord. Protein levels were significantly increased for the pro‐nflammatory cytokines IL‐1β (Figure 2(g); Psham vs. 14 d = 0.0072; *n* = 3 in the sham group, and *n* = 3 in the CCI group), IL‐6 (Figure 2(h); Psham vs. 14 d = 0.0009; *n* = 3 in the sham group, and *n* = 3 in the CCI group), and TNF‐α (Figure 2(i); Psham vs. 14 d = 0.0002; *n* = 3 in the sham group, and *n* = 3 in the CCI group) in the spinal cord of mice at postoperative day 14 compared to Sham group mice, consistent with the observed trend in microglial cell polarization.

Figure 2MIF induces microglial polarization in the spinal dorsal horn and neuroinflammation. (a) Representative images of Iba1 (green) in the spinal cord. (b) CCI‐induced fluorescence intensity increase of Iba1 in the spinal cord (scale bar = 50 μm). (c) Representative blots of CD68 and CD206 in the spinal cord. (d and e) Quantitative analyses of CD68 and CD206 in the spinal cord. (f) Representative blots of IL‐1β, IL‐6, and TNF‐α in the spinal cord. (g–i) Quantitative analyses of IL‐1β, IL‐6, and TNF‐α in the spinal cord. Data are expressed as the mean ± SD and represent the results of three independent experiments in three mice per group. ^∗∗^ *p*<0.01, ^∗∗∗^ *p*<0.001, and ^∗∗∗∗^ *p*<0.0001.

**Figure:**
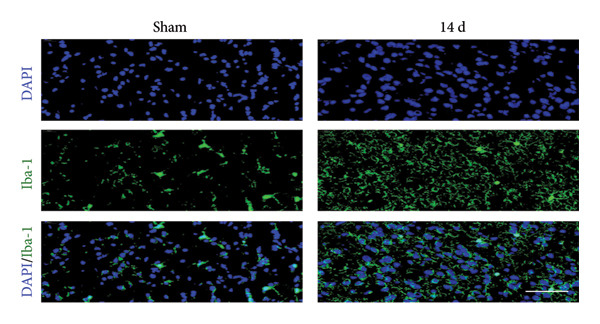
(a)

**Figure:**
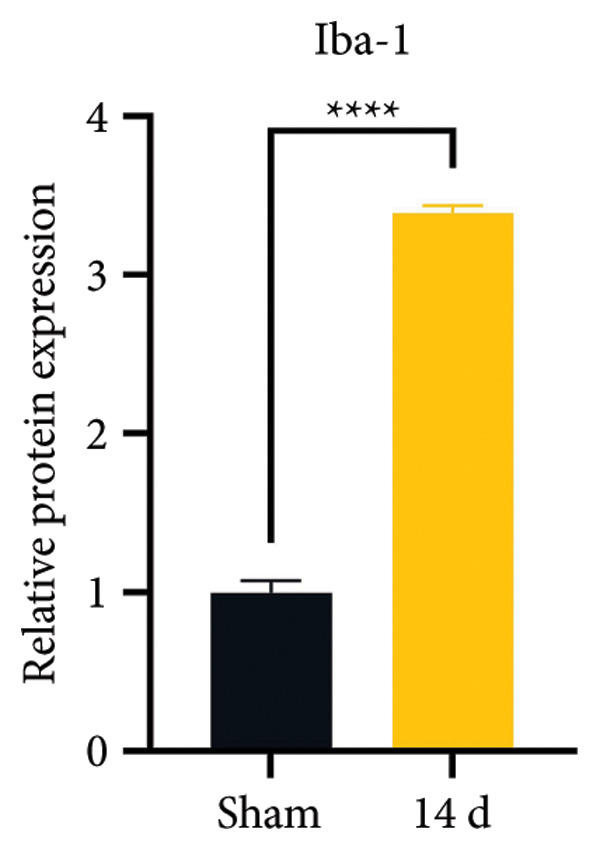
(b)

**Figure:**
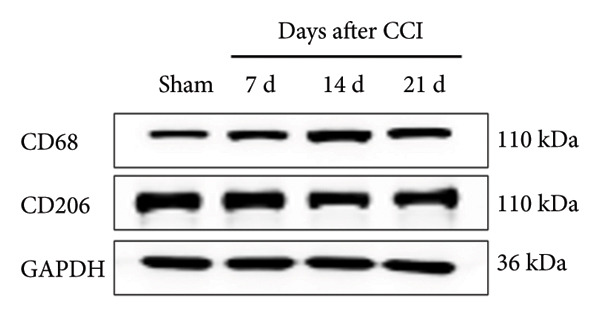
(c)

**Figure:**
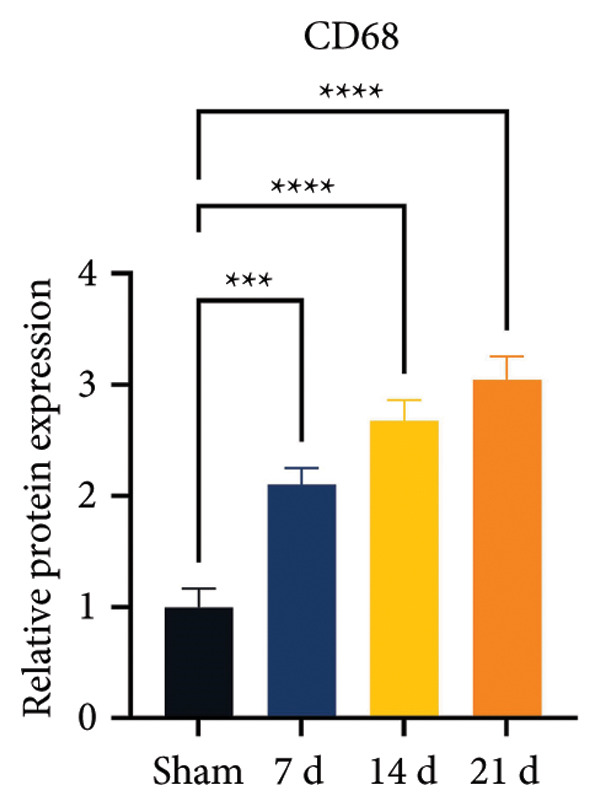
(d)

**Figure:**
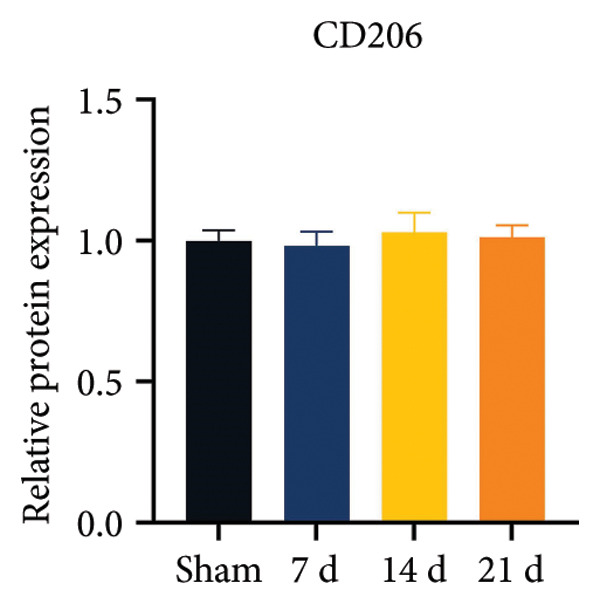
(e)

**Figure:**
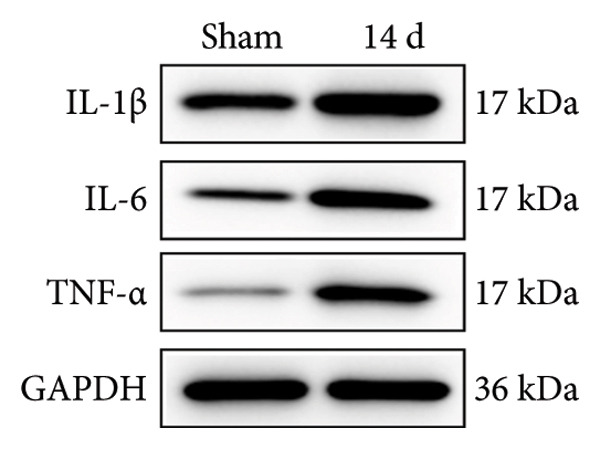
(f)

**Figure:**
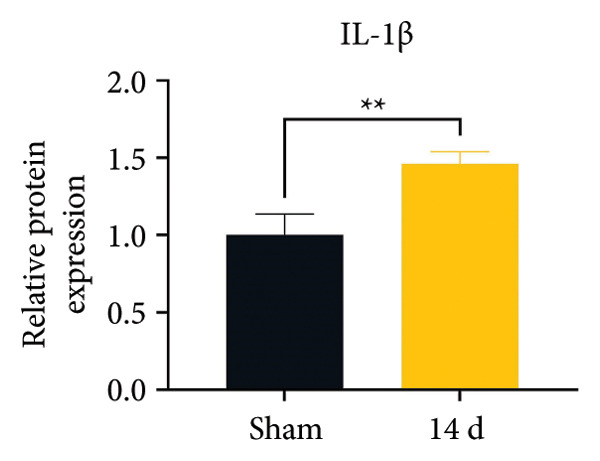
(g)

**Figure:**
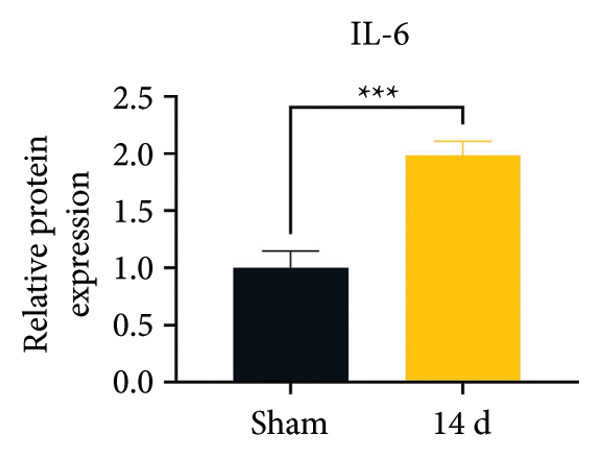
(h)

**Figure:**
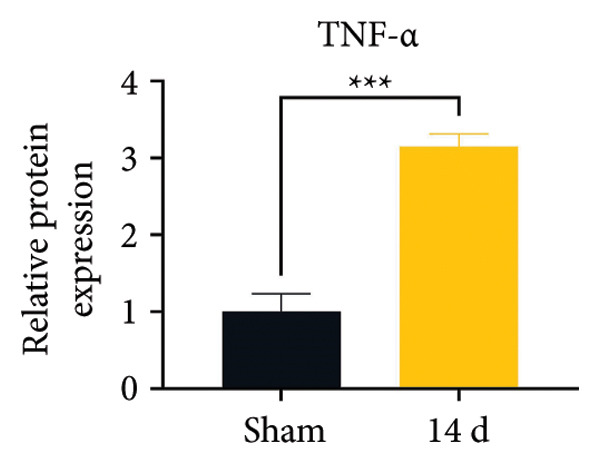
(i)

### 3.3. NLRP3 Inflammasome Activation and Neuronal Pyroptosis in Neuropathic Pain

Neuropathic pain increases the NLRP3 inflammasome levels in the spinal cord. Western blotting was performed to measure the levels of NLRP3 inflammasome‐related proteins (Figure 3(a)). Compared to the sham group, the expression levels of NLRP3 inflammasome‐related proteins in the spinal cord increased on postoperative day 14 (Figure 3(c):NLRP3: Psham vs. 14 d = 0.0023; Figure 3(b): Caspase 1: Psham vs. 14 d = 0.0102; *n* = 3 in the sham group, and *n* = 3 in the CCI group). The NLRP3 inflammasome regulates cell pyroptosis. The expression of the spinal cord pyroptosis‐related protein GSDMD‐N increased on postoperative day 14 compared to the Sham mice (Figure 3(d); GSDMD‐N: Psham vs. 14 d = 0.0013). The NF‐kB signaling pathway activates the NLRP3 inflammasome. Western blot analysis of P65 phosphorylation (p‐P65) showed an increased ratio of p‐P65 to P65 in the spinal cord at postoperative day 14, indicating the activation of the NF‐kB signaling pathway in neuropathic pain (Figure 3(e); p‐P65/P65: Psham vs. 14 d = 0.0140). The immunofluorescence results were consistent with the western blot results: the fluorescence intensity of NLRP3 and GSDMD significantly increased in the CCI group compared to the sham group on postoperative day 14 (Figures 3(f) and 3(g)).

Figure 3MIF activates the NLRP3 inflammasome to mediate neuronal pyroptosis in neuropathic pain. (a) Representative immunoblots of key pyroptosis‐related proteins (NLRP3, GSDMD‐N, and Caspase‐1) and phospho‐NF‐κB p65 (P‐P65), a marker of NF‐κB pathway activation, in the spinal cord. (b–e) Densitometric quantification of the protein levels of NLRP3, GSDMD‐N, Caspase‐1, and P‐P65, respectively. (f and g) Immunofluorescence analysis illustrating the expression levels of GSDMD (f) and NLRP3 (g) in the spinal cord across different experimental groups (Scale bar = 50 μm). All data are expressed as the mean ± SD from three independent experiments with three mice per group. ^∗^ *p* < 0.05, ^∗∗^ *p* < 0.01.

**Figure:**
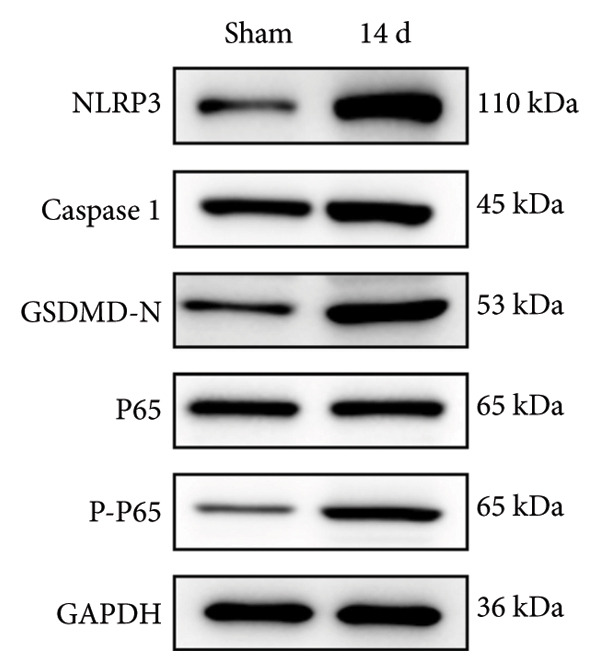
(a)

**Figure:**
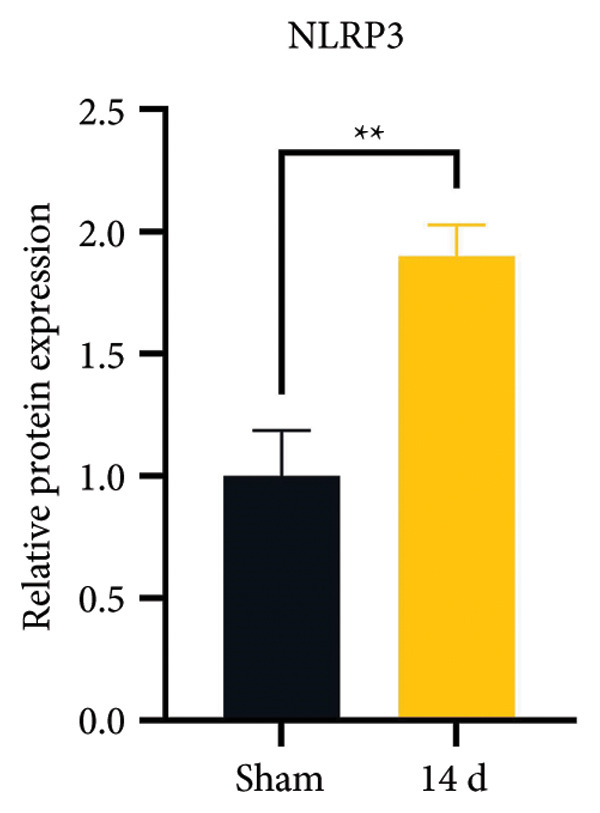
(b)

**Figure:**
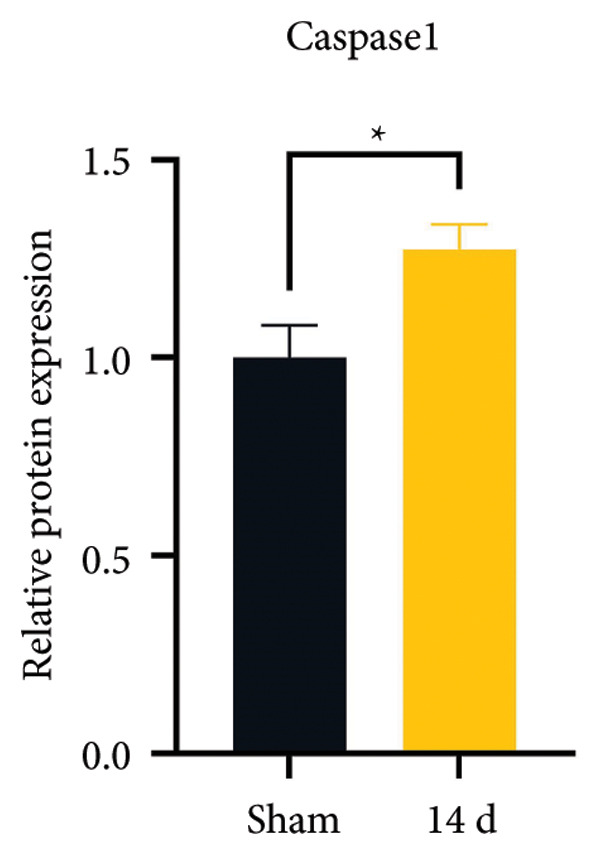
(c)

**Figure:**
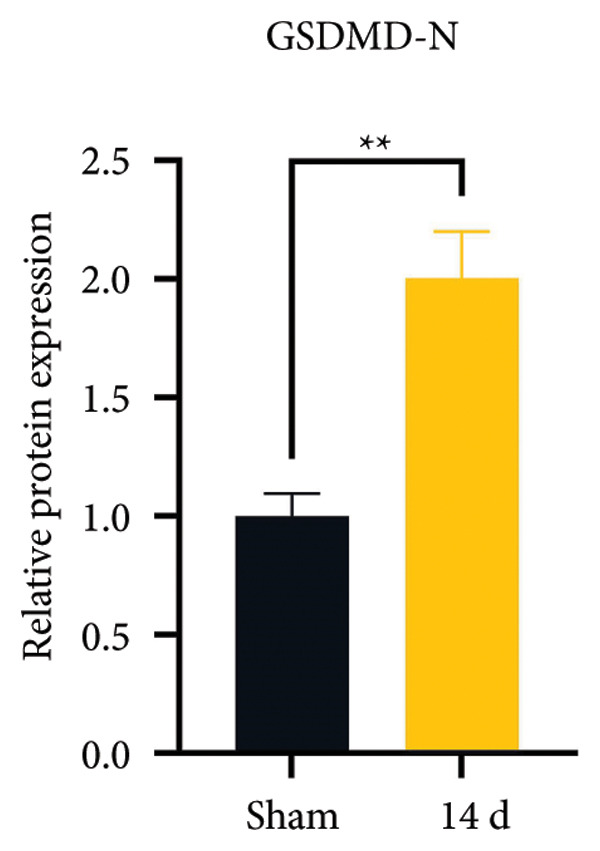
(d)

**Figure:**
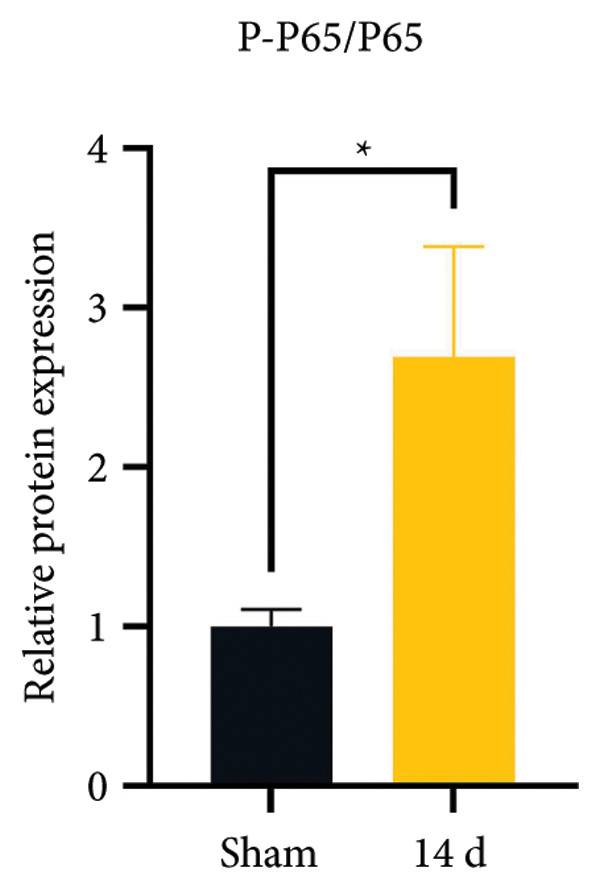
(e)

**Figure:**
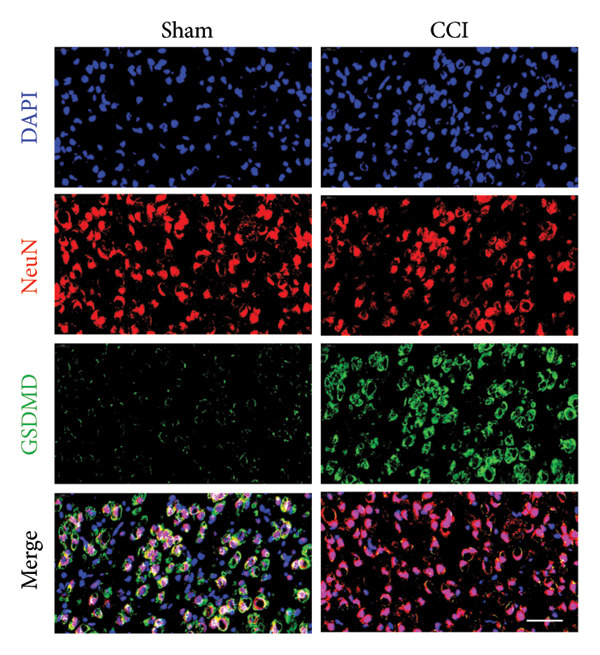
(f)

**Figure:**
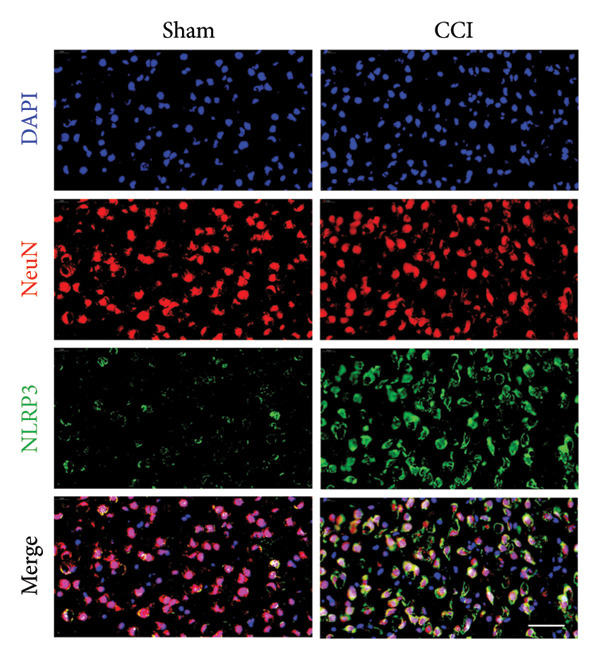
(g)

### 3.4. Inhibiting MIF Reduces Neuropathic Pain by Suppressing Neuronal Pyroptosis

We established a CCI+DMSO (dimethyl sulfoxide) group as the solvent control group to reduce experimental errors. Compared to the CCI+DMSO group, the CCI+ISO‐1 group showed improved pain hypersensitivity responses (ISO‐1 30 μg) (Figures 4(a) and 4(b); *n* = 10 in the sham group, and *n* = 10 in the CCI group). Intrathecal injection of the MIF inhibitor ISO‐1 decreased the expression of MIF (P _CCI+DMSO_ vs. _CCI+ISO-1_ = 0.0180), inflammasome‐related proteins NLRP3 (P _CCI+DMSO_ _vs._ _CCI+ISO-1_ = 0.0146; *n* = 3 in the sham group, and *n* = 3 in the CCI group), and caspase 1 (P _CCI+DMSO_ _vs._ _CCI+ISO-1_ = 0.0368; *n* = 3 in the sham group, and *n* = 3 in the CCI group) in the spinal cord of mice in the CCI+ISO‐1 group (Figure 4(d)). In addition to reducing inflammasome activation, cell pyroptosis was significantly improved, corresponding to decreased expression of the pyroptosis protein GSDMD‐N (Figure 4(d); GSDMD‐N: P _CCI+DMSO vs. CCI+ISO-1_ = 0.0244; *n* = 3 in the sham group, and *n* = 3 in the CCI group). We examined the protein levels of M1‐polarized microglial cells and inflammatory factors. The protein expression of CD68, IL‐1β, and TNF‐α in the CCI+ISO‐1 group decreased significantly compared to the CCI+DMSO group, but no significant difference was found in IL‐6 expression (Figures 4(e) and 4(f); CD68: P _CCI+DMSO vs. CCI+ISO-1_ = 0.0155; IL‐1β: P _CCI+DMSO vs. CCI+ISO-1_ = 0.0137; IL‐6: P _CCI+DMSO vs. CCI+ISO-1_ = 0.8584; TNF‐α: P _CCI+DMSO vs. CCI+ISO-1_ = 0.0438; *n* = 3 in the sham group, and *n* = 3 in the CCI group). The immunofluorescence results were consistent with the western blot results, showing a significant decrease in the fluorescence intensity of NLRP3 and GSDMD in the CCI+ISO‐1 group compared to the CCI+DMSO group on postoperative day 14 (Figures 4(g) and 4(h)).

Figure 4ISO‐1‐induced protection against neuronal pyroptosis caused by CCI in the spinal cord. (a) MWT and (b) TWL values were reduced following CCI surgery (14 days after), and intrathecal ISO‐1 administration attenuated these mechanical and thermal hypersensitivities in CCI surgery. (c) Representative Western blot images showing the protein levels of MIF, P‐P65, and pyroptosis‐related proteins (NLRP3, GSDMD‐N, and Caspase‐1) in the spinal cord. (d) Quantitative analysis of the protein levels presented in (c). (e) Representative immunoblots of the microglial marker CD68 and proinflammatory cytokines (IL‐1β, IL‐6, and TNF‐α). (f) Quantitative analysis of the protein levels presented in (e). (g, h) Double‐label immunofluorescence staining for GSDMD (g) and NLRP3 (h) with the neuronal marker NeuN in the spinal cord (Scale bar = 50 μm). Data are expressed as the mean ± SD (*n* = 3 independent experiments with three mice per group). ^∗^ *p* < 0.05, ^∗∗^ *p* < 0.01.

**Figure:**
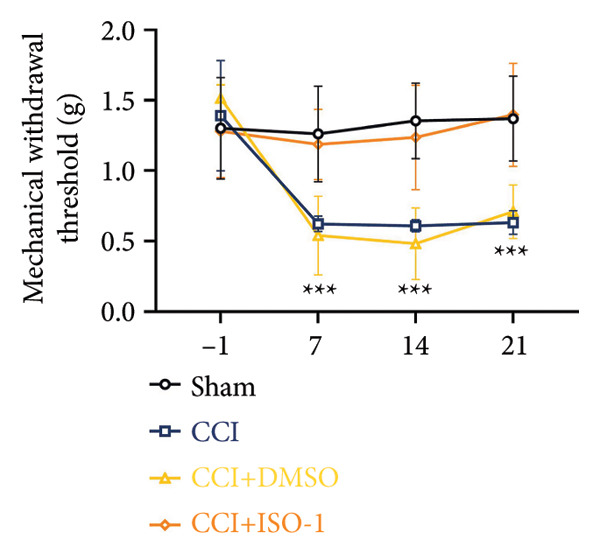
(a)

**Figure:**
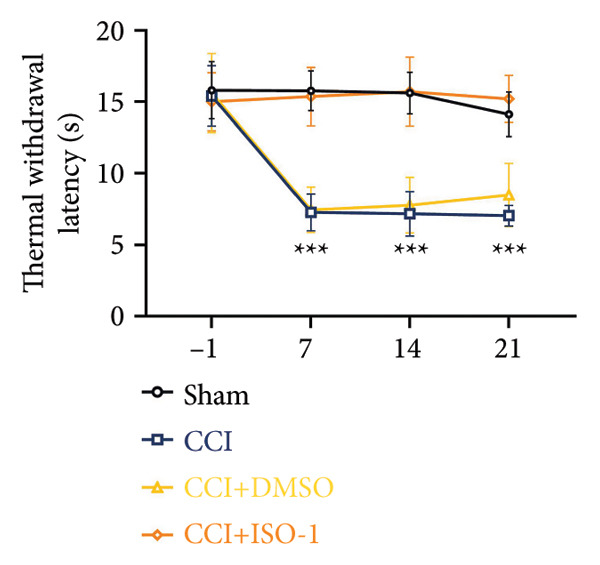
(b)

**Figure:**
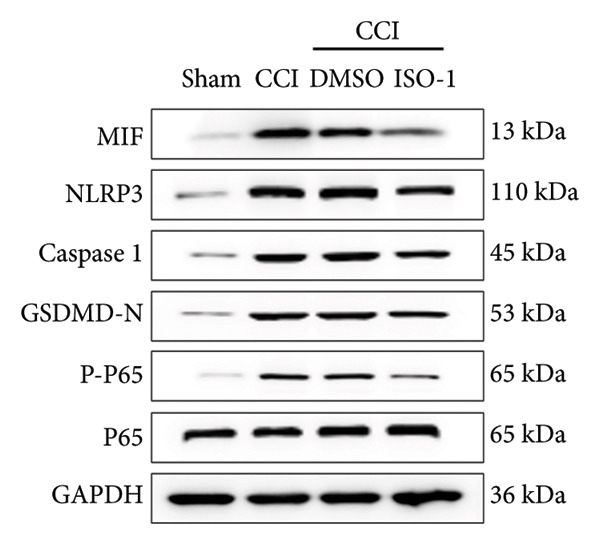
(c)

**Figure:**
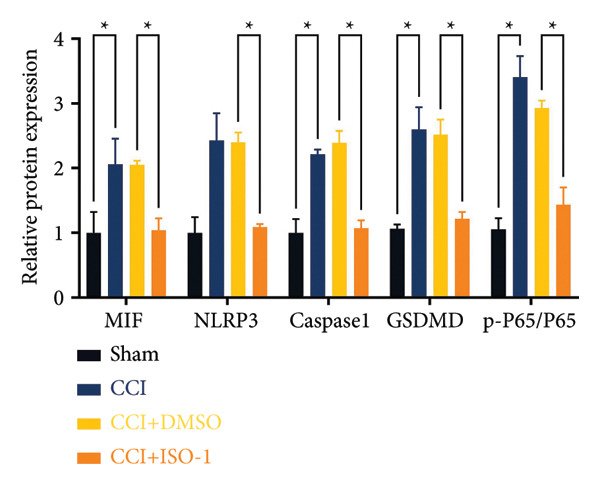
(d)

**Figure:**
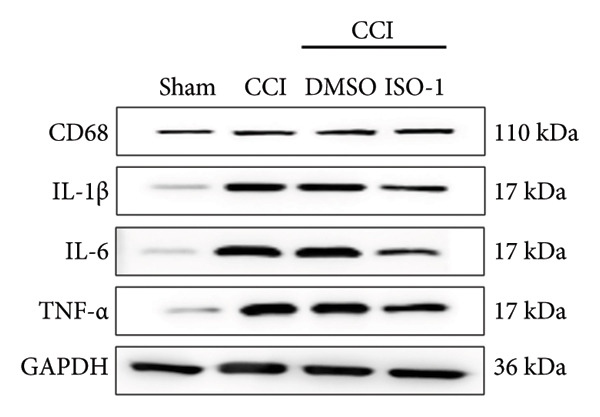
(e)

**Figure:**
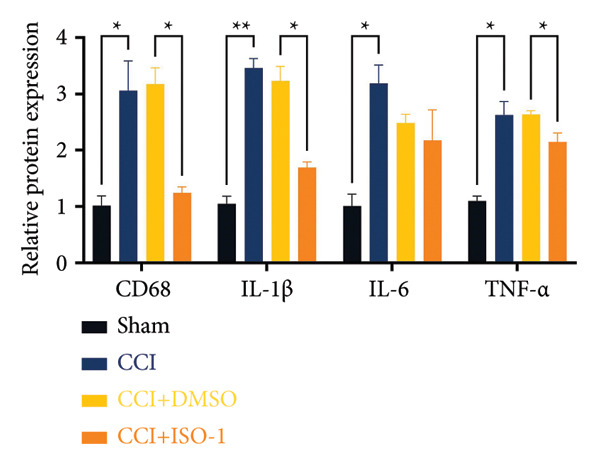
(f)

**Figure:**
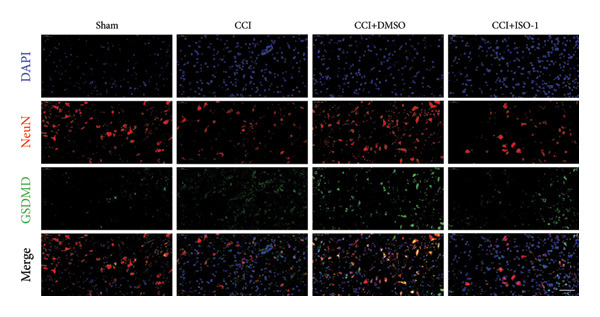
(g)

**Figure:**
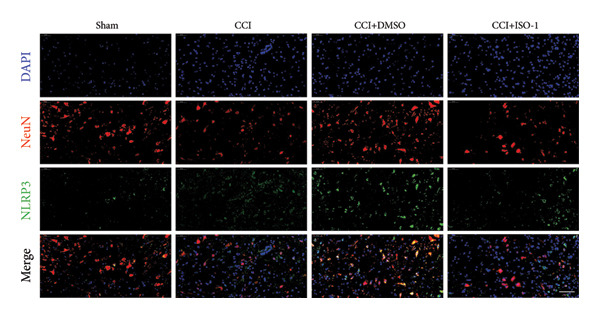
(h)

### 3.5. NF‐κB Inhibition Attenuates NLRP3‐Mediated Pyroptosis in Neuropathic Pain

Studies on the NF‐κB‐NLRP3 pathway have provided insights. To elucidate the mechanism by which MIF regulates NLRP3‐mediated neuronal pyroptosis in neuropathic pain, we used PDTC to inhibit the NF‐κB signaling pathway. The CCI group (21 days of CCI induction) was treated with 50 mg/kg NF‐κB p65 inhibitor PDTC (once a day for 21 days via intraperitoneal injection). Pain hypersensitivity responses were ameliorated in the CCI+PDTC group compared to the CCI group (Figures 5(a) and 5(b); *n* = 10 in the sham group, and *n* = 10 in the CCI group). Following intraperitoneal injection of the NF‐κB inhibitor PDTC, the expression of NLRP3 decreased in the spinal cord of mice in the CCI+PDTC group compared to that in the CCI group; however, we found no statistically significant difference in Caspase 1 expression between the two groups (Figure 5(d); NLRP3: P _CCI vs. CCI+ PDTC_ = 0.0161; Caspase 1: P _CCI vs. CCI+ PDTC_ = 0.0980; *n* = 3 in the sham group, and *n* = 3 in the CCI group). In addition to reducing inflammasome activation, neuron pyroptosis was significantly improved, corresponding to decreased expression of the pyroptosis protein GSDMD‐N (Figure 5(d); GSDMD‐N: P _CCI+DMSO vs. CCI+ISO-1_ = 0.0188; *n* = 3 in the sham group, and *n* = 3 in the CCI group). Examination of the protein levels of M1‐polarized microglial cells and inflammatory factors showed that the expression of IL‐1β, IL‐6, and TNF‐α in the CCI+PDTC group significantly decreased compared to the CCI group (Figures 5(e) and 5(f); CD68: P _CCI+DMSO vs. CCI+ISO-1_ = 0.0249; IL‐1β: P _CCI+DMSO vs. CCI+ISO-1_ = 0.0326; IL‐6: P _CCI+DMSO vs. CCI+ISO-1_ = 0.0060; TNF‐α: P _CCI+DMSO vs. CCI+ISO-1_ = 0.0248; *n* = 3 in the sham group, and *n* = 3 in the CCI group).

Figure 5PDTC‐induced protection against neuronal pyroptosis caused by CCI in the spinal cord. (a) MWT and (b) TWL values were reduced following CCI surgery, and intrathecal PDTC administration attenuated these mechanical and thermal hypersensitivities in CCI surgery. (c) Representative Western blot images depicting the expression of pyroptosis‐associated proteins (NLRP3, GSDMD‐N, and Caspase‐1) and phospho‐NF‐κB p65 (P‐P65) in the spinal cord. (d) Quantitative analysis of the protein levels shown in (c). (e) Representative immunoblots for CD68 and inflammatory cytokines (IL‐1β, IL‐6, and TNF‐α). (f) Quantitative analysis of the protein levels shown in (e). Data are presented as the mean ± SD from three independent experiments with three mice per group. ^∗^ *p* < 0.05, ^∗∗^ *p* < 0.01, and ^∗∗∗^ *p* < 0.001.

**Figure:**
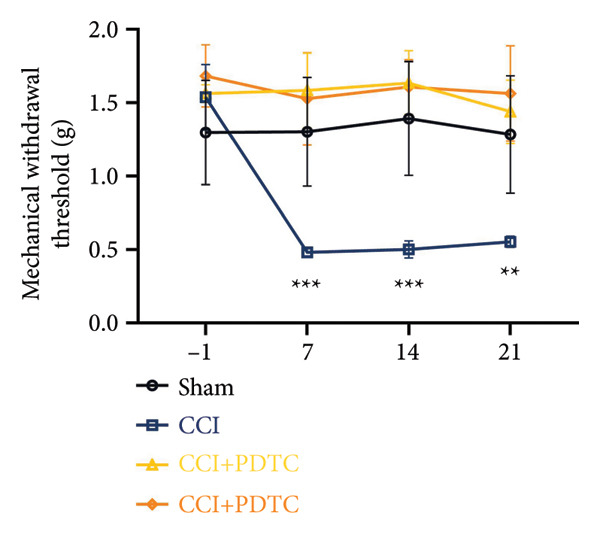
(a)

**Figure:**
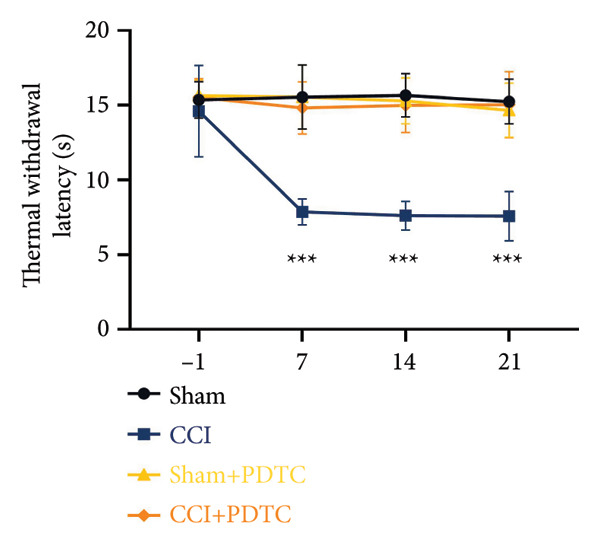
(b)

**Figure:**
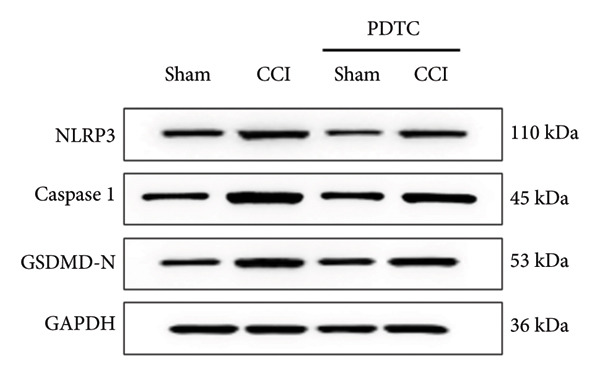
(c)

**Figure:**
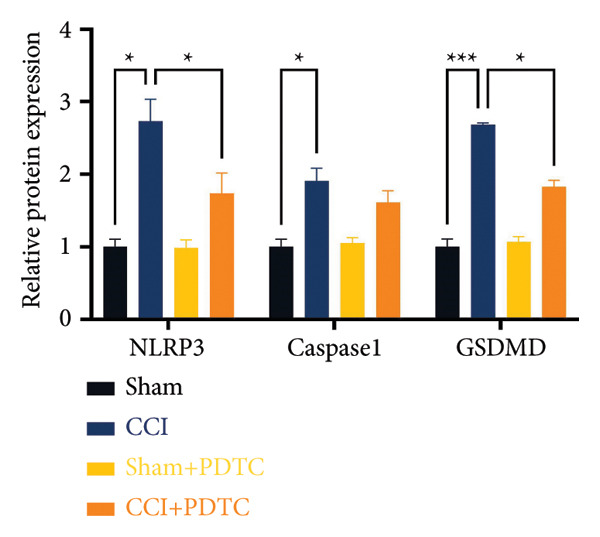
(d)

**Figure:**
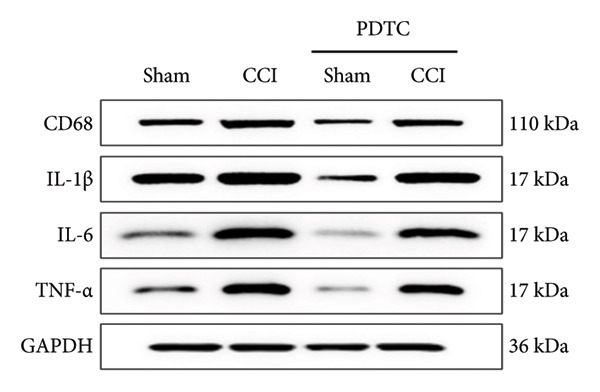
(e)

**Figure:**
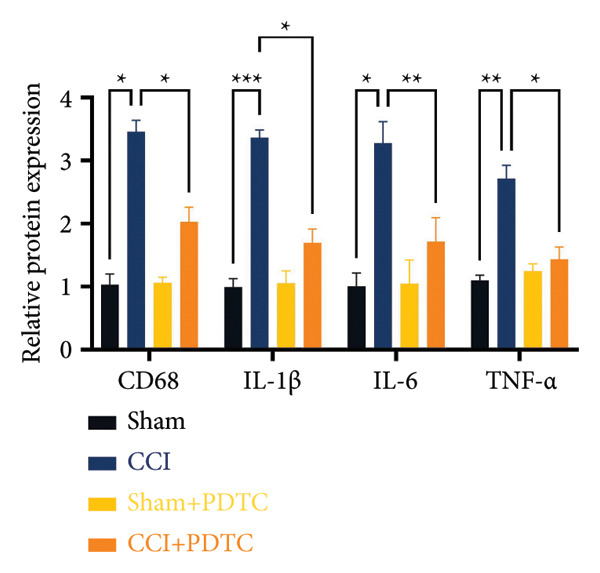
(f)

## 4. Discussion

Neuropathic pain represents a debilitating clinical condition that profoundly compromises the quality of life of affected individuals [19]. While spinal neuronal pyroptosis and microglial polarization have been established as key contributors to its pathogenesis, the upstream regulatory triggers initiating these processes remain largely elusive. Our study elucidates a novel mechanism whereby Macrophage MIF exacerbates neuropathic pain by facilitating NLRP3 inflammasome‐mediated neuronal pyroptosis. The principal findings supporting this conclusion are as follows: First, neuropathic pain was found to upregulate MIF expression within the spinal cord, where it colocalized predominantly with neuronal markers. Second, MIF promotes the polarization of microglia toward a proinflammatory M1 phenotype, concomitant with an increased release of inflammatory cytokines, including IL‐1β, IL‐6, and TNF‐α. Third, the expression of pyroptosis‐executing proteins downstream of the NLRP3 inflammasome was significantly elevated in the spinal cords of neuropathic pain models. Fourth, pharmacological inhibition of MIF with ISO‐1 attenuated NLRP3 inflammasome–induced neuronal pyroptosis, as evidenced by reduced levels of pyroptosis‐related proteins, suppressed microglial M1 polarization, and diminished neuroinflammation, ultimately leading to the alleviation of mechanical allodynia and thermal hyperalgesia. Finally, inhibition of the NF‐κB pathway with PDTC similarly ameliorated pain hypersensitivity by suppressing the NLRP3 inflammasome‐pyroptosis axis. In summary, our findings demonstrate that MIF contributes to the development of neuropathic pain by activating the NF‐κB signaling pathway, which in turn induces NLRP3 inflammasome‐dependent neuronal pyroptosis and promotes proinflammatory microglial polarization.

Previous MIF exacerbates neuropathic pain and mechanical or thermal hyperalgesia caused by spinal cord or nerve injury [20]. As resident immune cells of the central nervous system, microglia are rapidly activated in response to signals, exhibiting classical (M1) and alternative (M2) activation phenotypes and releasing proinflammatory or anti‐inflammatory factors [21]. In neuropathic pain, spinal microglia are rapidly activated and undergo M1 polarization, releasing large numbers of proinflammatory factors. However, the source of MIF remains unclear. Our study indicates that neuropathic pain increases the immunofluorescence intensity of MIF, with its expression progressively increasing over time and peaking on postoperative day 14. MIF expression co‐localizes with neuronal markers in the spinal cord rather than with microglial or astrocytic markers. While laminar distribution was not quantitatively analyzed, our qualitative assessment suggests that MIF/pyroptosis occurs broadly across nociceptive processing regions. Consistent with previous studies, our research confirms that MIF promotes M1 polarization of microglia, increasing protein expression of proinflammatory factors TNF‐α, IL‐1β, and IL‐6. Our findings suggest that MIF is crucial in regulating microglial polarization in spinal neurons.

In addition to their critical role in immune inflammation, microglia regulate neuronal homeostasis [22]. Although numerous studies have investigated the pathogenesis of neuropathic pain involving microglia, the role of spinal neurons in the onset of neuropathic pain remains unclear. Pathological physiological changes in spinal neurons trigger the pathophysiological processes of nociceptive hypersensitivity reactions [23]. For example, changes in programmed cell death (e.g., autophagy and ferroptosis) have been identified, and researchers have elucidated various necroptotic mechanisms in pain‐sensing spinal neurons; however, the necroptotic mechanism of neuropathic pain in spinal neurons remains unclear [24, 25]. Characteristics of necroptosis include activating caspase 1 and the cleavage of GSDMD by caspase 1 into GSDMD‐N, leading to pore formation and increasing mature IL‐1β secretion. NLRP3 inflammasome activation induces pyroptosis, which is involved in inflammatory disease. Our study’s protein imprint analysis and immunofluorescence data revealed increased expression of spinal neuron pyroptosis‐related proteins, including GSDMD‐ and NLRP3‐related proteins, in CCI mice. This finding indicates that NLRP3 inflammasome activation mediates pyroptosis in spinal neurons.

Activation of the NLRP3 inflammasome requires MIF [26]. The mechanism by which MIF regulates the activation of NLRP3 remains unclear. However, MIF exhibits molecular chaperone‐like activity with NLRP3‐related proteins. MIF exhibits molecular chaperone‐like activity through its interactions with proteins associated with the NLRP3 inflammasome. Functioning as an ATP‐independent chaperone, MIF prevents the misfolding of mutant superoxide dismutase, a process whose pathological accumulation is implicated in the progression of neurodegenerative diseases [27]. Therefore, MIF is a companion of the NLRP3 inflammasome, potentially stabilizing NLRP3 inflammasome proteins or promoting their function [28]. ISO‐1 is the most commonly used inhibitor of MIF. Our study shows that intrathecal administration of ISO‐1 reduces the expression levels of proinflammatory factors IL‐1β, IL‐6, and TNF‐α induced by microglial M1 polarization, alleviating neuropathic pain. Furthermore, our results demonstrate that intrathecal administration of the MIF inhibitor ISO‐1 effectively attenuates NLRP3 inflammasome‐mediated neuronal pyroptosis. This pharmacological inhibition was characterized by a significant reduction in the expression of key inflammasome components, namely, NLRP3 and caspase‐1. These findings collectively indicate that MIF‐induced microglial polarization and NLRP3 inflammasome‐driven neuronal pyroptosis in neuropathic pain represent reversible pathological processes that can be therapeutically targeted by MIF inhibition.

NF‐κB regulates inflammatory response, participating in the transcription of chemokines and inflammatory factors [29]. Under normal conditions, NF‐κB remains inactive by binding with inhibitory protein IκBα in the cytoplasm. Upon cellular stimulation, signaling cascades induce phosphorylation of IκB by IκB kinase, leading to the degradation of phosphorylated IκB (p‐IκB) by the ubiquitin‐proteasome system [30]. After IκB degradation, NF‐κB dissociates from IκBα, becomes active, translocates freely into the cell nucleus, binds to relevant DNA sequences, and regulates the gene transcription, including NLRP3 [31]. Consistent with previous studies, we found upregulation of p‐P65 expression in the spinal cord of CCI mice. Treatment with the NF‐kB inhibitor PDTC inhibited the expression of p‐P65, resulting in reduced NLRP3 inflammasome–induced neuronal pyroptosis and inhibited expression of microglial polarization–induced inflammatory factors. These findings suggest that inhibiting NF‐κB is a potential therapeutic target for alleviating neuropathic pain by reducing NLRP3 inflammasome activation.

This study had several limitations. First, we focused specifically on the effect of MIF inhibitors on the NLRP3 inflammasome and did not investigate the differential effects of MIF on its molecular components, including NLRP3 and caspase 1. Secondly, this preliminary study did not fully elucidate the specific cellular mechanisms underlying the inhibitory effect of NF‐kB inhibitors on microglial polarization; thus, further research is needed. Although our data show spatial and temporal correlation between neuronal MIF upregulation, microglial M1 polarization, and NLRP3 activation, causal linkage requires further validation.

**Figure 6:**
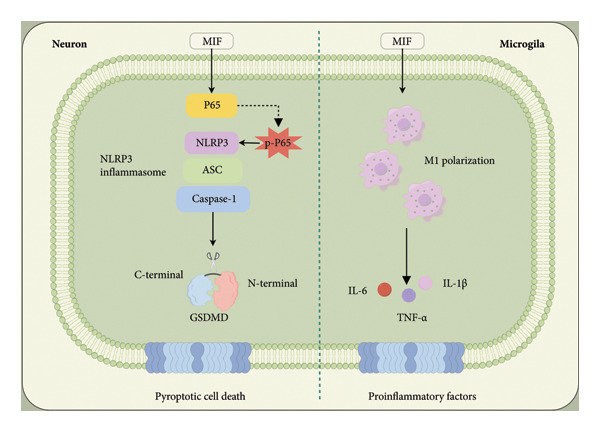
Graphical Abstract. MIF promotes neuronal pyroptosis mediated by the NLRP3 inflammasome and microglial polarization through the NF‐κB signaling pathway in neuropathic pain.

In summary, our findings collectively demonstrate that MIF exacerbates neuropathic pain by activating the NF‐κB/NLRP3 pathway, leading to neuronal pyroptosis and microglial M1 polarization. This proposed mechanism is summarized in Figure 6, which illustrates the interplay between MIF, NF‐κB signaling, NLRP3 inflammasome activation, and subsequent neuroinflammation in the spinal cord. Targeting this pathway may offer novel therapeutic strategies for neuropathic pain management.

## Conflicts of Interest

The authors declare no conflicts of interest.

## Author Contributions

Feng Zhou conceived, designed, and did statistical analysis and editing of manuscript, and is responsible for the integrity of the research.

Yue Tian, Wei Liao, Qingling Ma, and Han Bao did data collection and manuscript writing.

Fanqing Meng and Jingjing Jiang did the review and the final approval of the manuscript.

## Funding

This research was funded by the Jinan Science and Technology Bureau (Grant/Award Number: 202134071) and the Shandong Medical Health Science and Technology Project (Grant/Award Number: 202318001388).

## Data Availability

The data that support the findings of this study are available on request from the corresponding author. The data are not publicly available due to privacy or ethical restrictions.
