# Correction to “MIF‐Mediated NLRP3 Inflammasome‐Dependent Pyroptosis in Spinal Neurons and Microglial Polarization Facilitate Neuropathic Pain Progression”

**DOI:** 10.1155/anrp/9818954

**Published:** 2026-05-26

F. Zhou, Y. Tian, W. Liao, et al., “MIF‐Mediated NLRP3 Inflammasome‐Dependent Pyroptosis in Spinal Neurons and Microglial Polarization Facilitate Neuropathic Pain Progression,” *Anesthesiology Research and Practice*, vol. 2025 (2025). https://doi.org/10.1155/anrp/6624776.

In the article titled “MIF‐Mediated NLRP3 Inflammasome‐Dependent Pyroptosis in Spinal Neurons and Microglial Polarization Facilitate Neuropathic Pain Progression,” there were errors in multiple figures. These errors are summarised and corrected below:

Figure 3: The merged images of the “Sham” and “CCI” groups were originally placed in the reverse order. This error was introduced by the authors during figure assembly and Figure 3 should be corrected as follows:

FIGURE 3MIF activates the NLRP3 inflammasome to mediate neuronal pyroptosis in neuropathic pain. (a) Representative immunoblots of key pyroptosis‐related proteins (NLRP3, GSDMD‐N, and Caspase‐1) and phospho‐NF‐κB p65 (P‐P65), a marker of NF‐κB pathway activation, in the spinal cord. (b–e) Densitometric quantification of the protein levels of NLRP3, GSDMD‐N, Caspase‐1, and P‐P65, respectively. (f and g) Immunofluorescence analysis illustrating the expression levels of GSDMD (f) and NLRP3 (g) in the spinal cord across different experimental groups (scale bar = 50 μm). All data are expressed as the mean ± SD from three independent experiments with three mice per group. ^∗^ *p* < 0.05 and ^∗∗^ *p* < 0.01.

**Figure:**
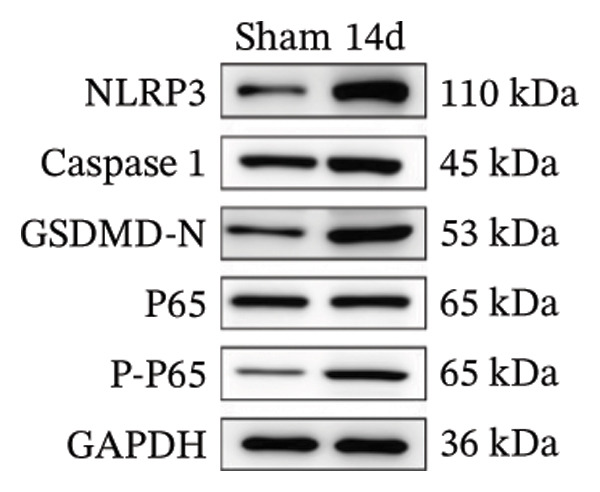
(a)

**Figure:**
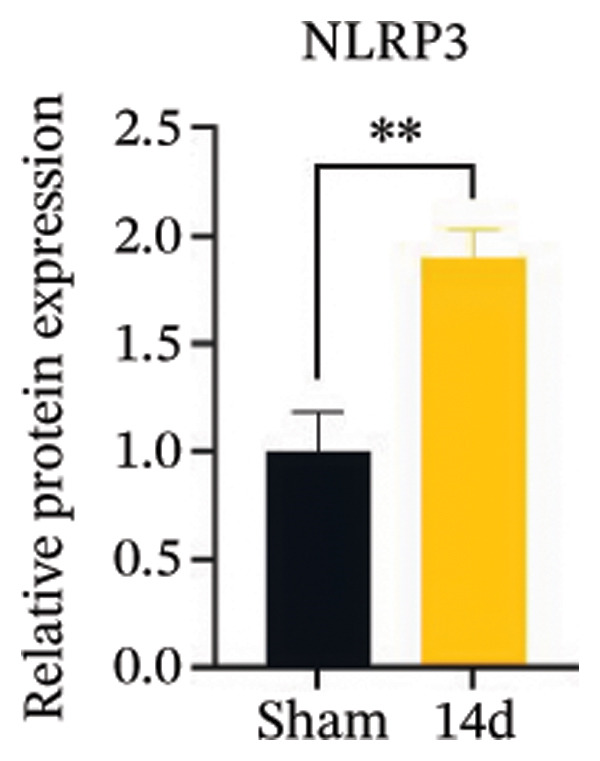
(b)

**Figure:**
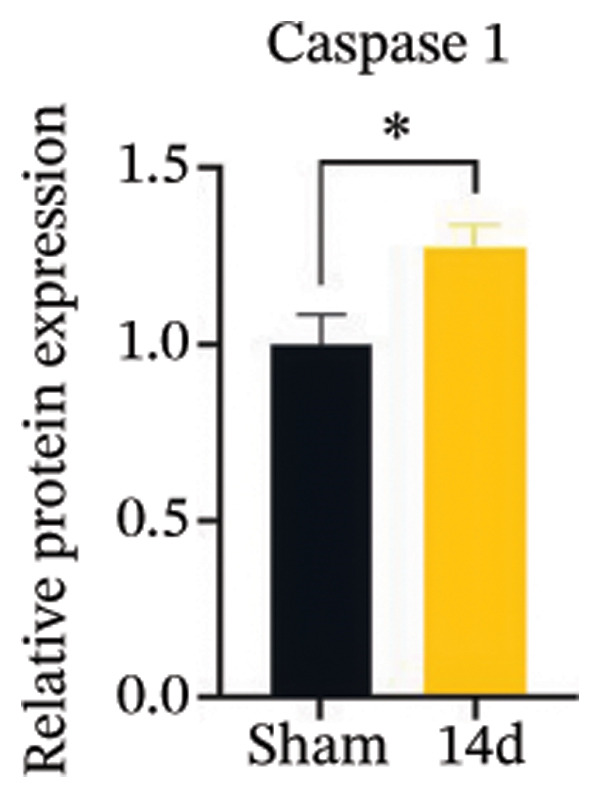
(c)

**Figure:**
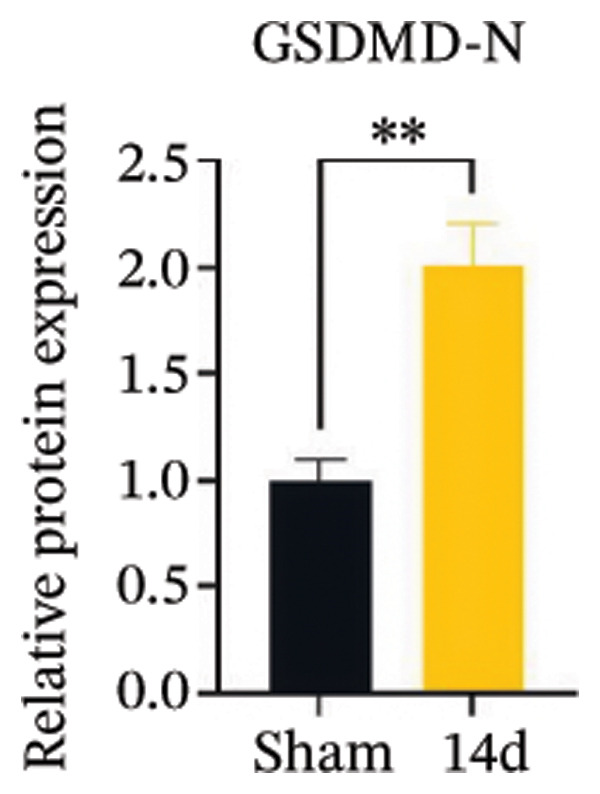
(d)

**Figure:**
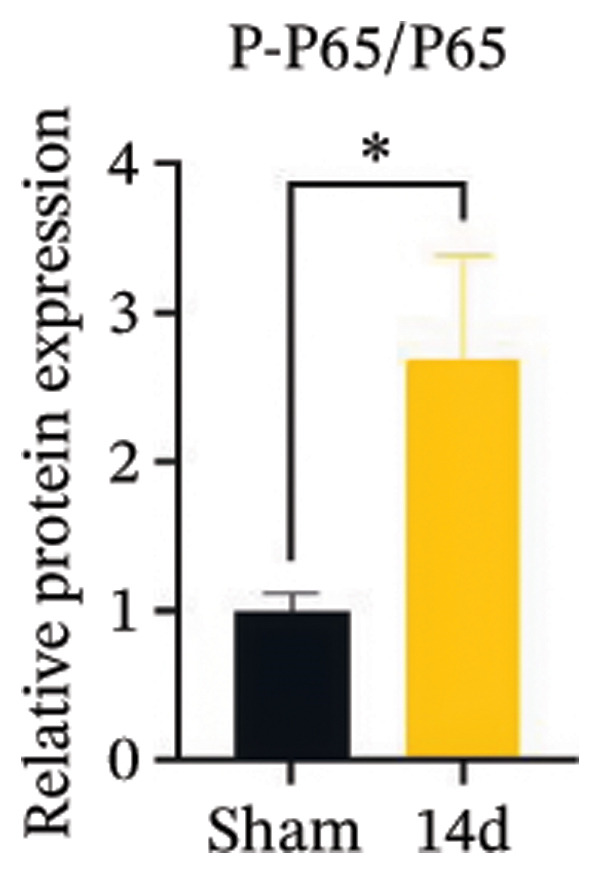
(e)

**Figure:**
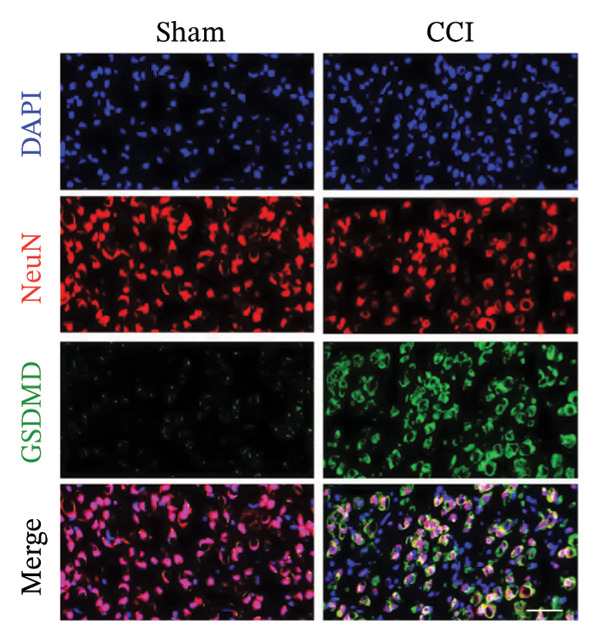
(f)

**Figure:**
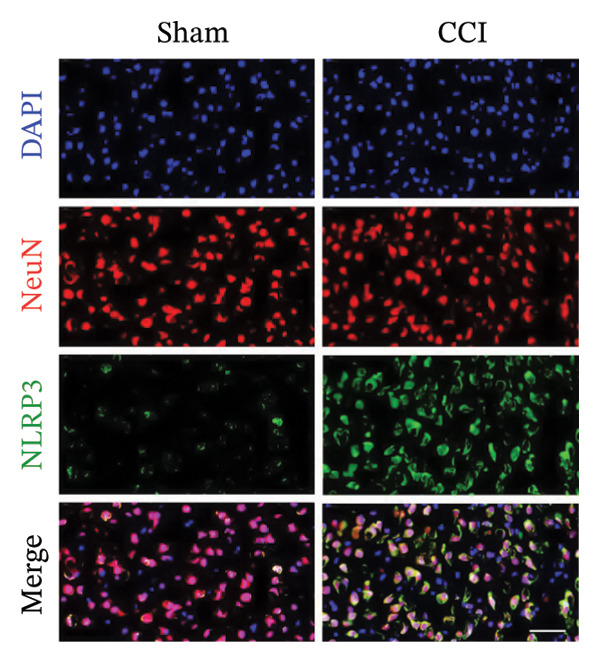
(g)

Figure 4: The immunofluorescence staining images shown in panel (g) were inadvertently duplicated in panel (h). This error was introduced by the publisher during the production of the article and Figure 4 should be corrected as follows:

FIGURE 4ISO‐1‐induced protection against neuronal pyroptosis caused by CCI in the spinal cord. (a) MWT and (b) TWL values were reduced following CCI surgery (14 days after), and intrathecal ISO‐1 administration attenuated these mechanical and thermal hypersensitivities in CCI surgery. (c) Representative Western blot images showing the protein levels of MIF, P‐P65, and pyroptosis‐related proteins (NLRP3, GSDMD‐N, and Caspase‐1) in the spinal cord. (d) Quantitative analysis of the protein levels presented in (c). (e) Representative immunoblots of the microglial marker CD68 and proinflammatory cytokines (IL‐1β, IL‐6, and TNF‐α). (f) Quantitative analysis of the protein levels presented in (e). (g, h) Double‐label immunofluorescence staining for GSDMD (g) and NLRP3 (h) with the neuronal marker NeuN in the spinal cord (scale bar = 50 μm). Data are expressed as the mean ± SD (*n* = 3 independent experiments with three mice per group). ^∗^ *p* < 0.05 and ^∗∗^ *p* < 0.01.

**Figure:**
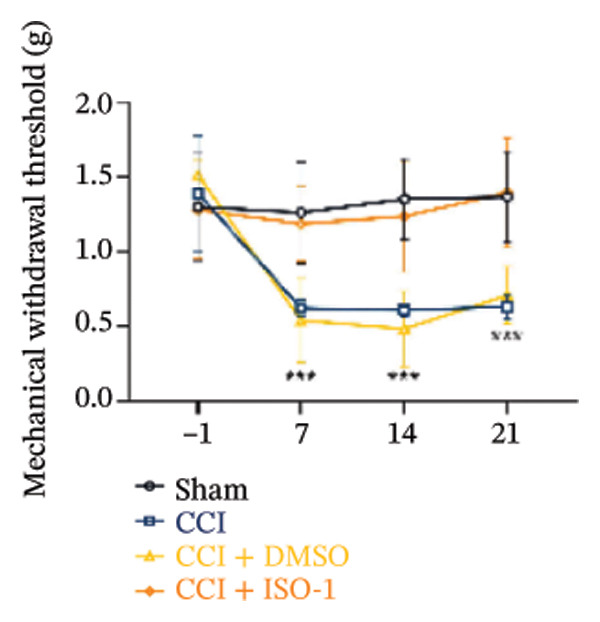
(a)

**Figure:**
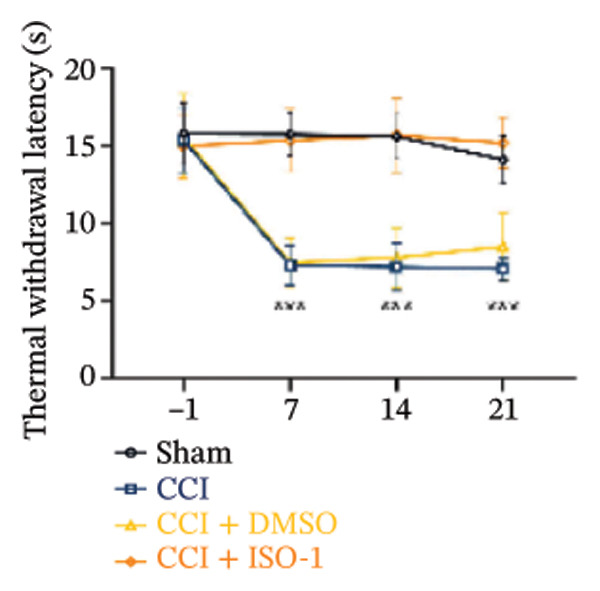
(b)

**Figure:**
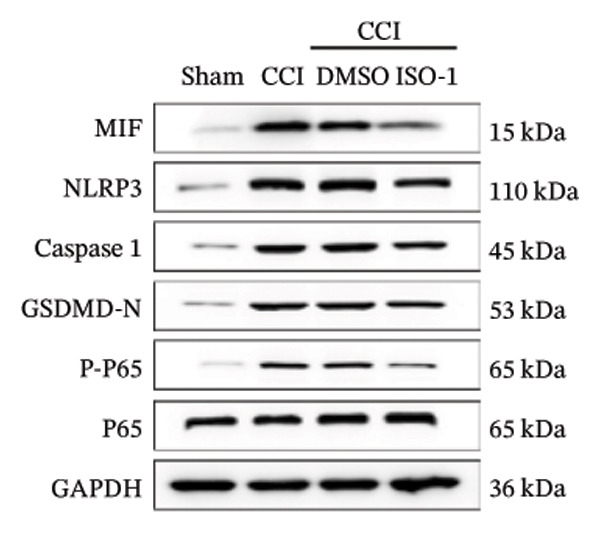
(c)

**Figure:**
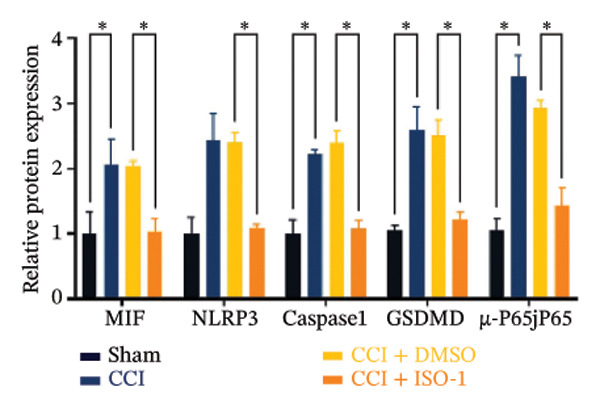
(d)

**Figure:**
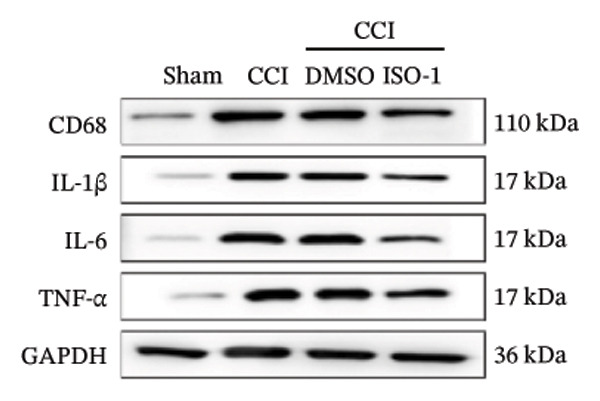
(e)

**Figure:**
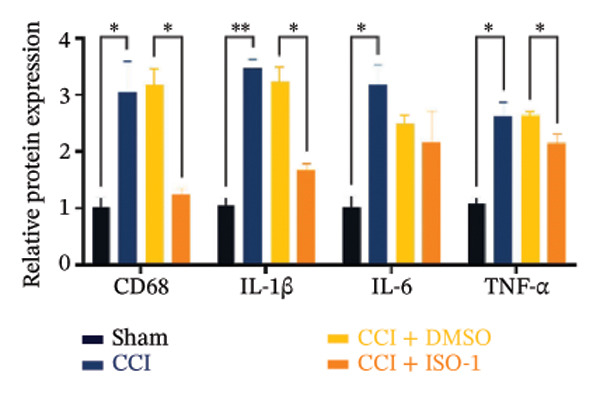
(f)

**Figure:**
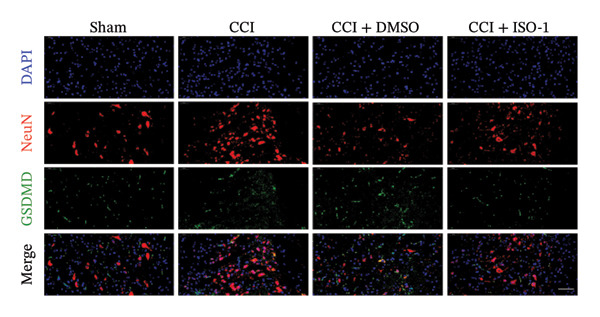
(g)

**Figure:**
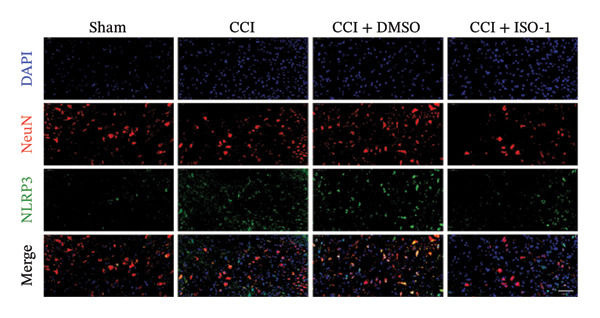
(h)

Figure 5: The bands representing the expression of CD68 in Figure 5E were accidently used to denote the expression of NLRP3 in Figure 5C. This error was introduced by the authors during the assembly of the figure and the corrected Figure 5 is presented below:

FIGURE 5PDTC‐induced protection against neuronal pyroptosis caused by CCI in the spinal cord. (a) MWT and (b) TWL values were reduced following CCI surgery, and intrathecal PDTC administration attenuated these mechanical and thermal hypersensitivities in CCI surgery. (c) Representative Western blot images depicting the expression of pyroptosis‐associated proteins (NLRP3, GSDMD‐N, and Caspase‐1) and phospho‐NF‐κB p65 (P‐P65) in the spinal cord. (d) Quantitative analysis of the protein levels shown in (c). (e) Representative immunoblots for CD68 and inflammatory cytokines (IL‐1β, IL‐6, and TNF‐α). (f) Quantitative analysis of the protein levels shown in (e). Data are presented as the mean ± SD from three independent experiments with three mice per group. ^∗^ *p* < 0.05, ^∗∗^ *p* < 0.01, and^∗∗∗^ *p* < 0.001.

**Figure:**
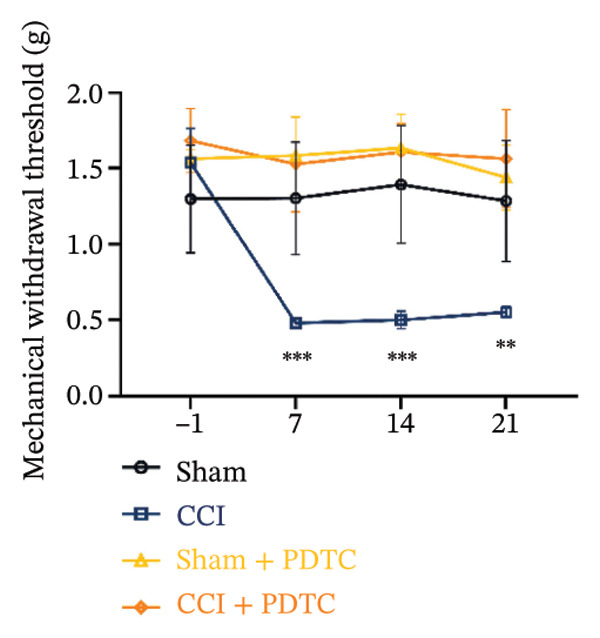
(a)

**Figure:**
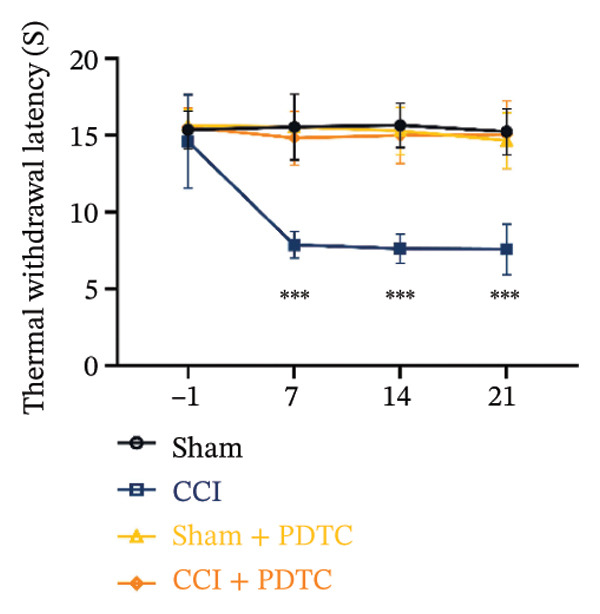
(b)

**Figure:**
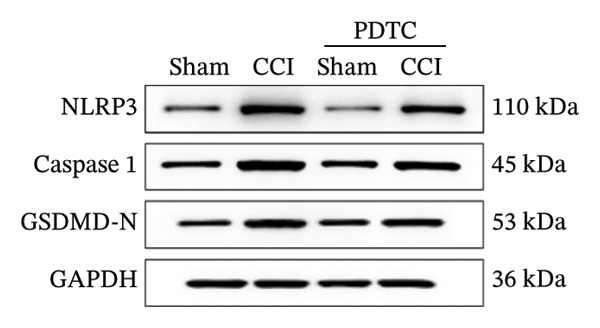
(c)

**Figure:**
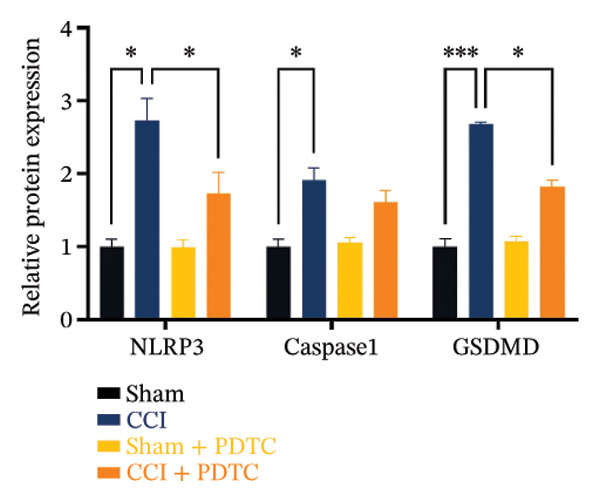
(d)

**Figure:**
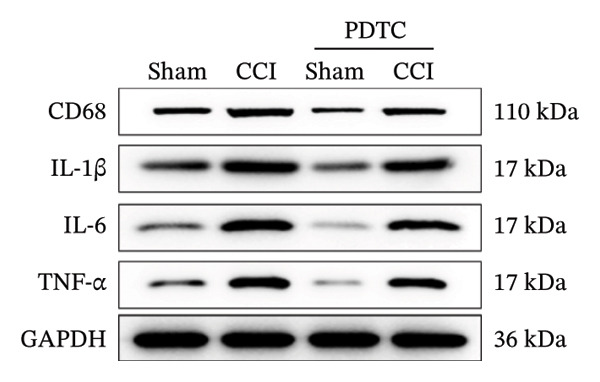
(e)

**Figure:**
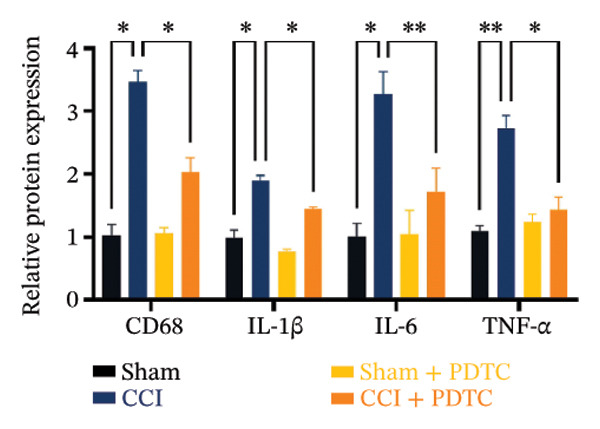
(f)

We apologise for these errors.

